# AsHSP26.8a, a creeping bentgrass small heat shock protein integrates different signaling pathways to modulate plant abiotic stress response

**DOI:** 10.1186/s12870-020-02369-5

**Published:** 2020-04-28

**Authors:** Xinbo Sun, Junfei Zhu, Xin Li, Zhigang Li, Liebao Han, Hong Luo

**Affiliations:** 1 Key Laboratory of Crop Growth Regulation of Hebei Province, College of Agronomy, Hebei Agricultural University, Baoding, Hebei 071001 People’s Republic of China; 2 Department of Genetics and Biochemistry, Clemson University, 110 Biosystems Research Complex, Clemson, SC 29634 USA; 3 Turfgrass Research Institute, Beijing Forestry University, Beijing, 100083 People’s Republic of China

**Keywords:** Creeping bentgrass, AsHSP26.8a, ABA, Abiotic stress, Small heat shock protein

## Abstract

**Background:**

Small heat shock proteins (sHSPs) are critical for plant response to biotic and abiotic stresses, especially heat stress. They have also been implicated in various aspects of plant development. However, the acting mechanisms of the sHSPs in plants, especially in perennial grass species, remain largely elusive.

**Results:**

In this study, *AsHSP26.8a*, a novel chloroplast-localized sHSP gene from creeping bentgrass (*Agrostis stolonifera* L.) was cloned and its role in plant response to environmental stress was studied. *AsHSP26.8a* encodes a protein of 26.8 kDa. Its expression was strongly induced in both leaf and root tissues by heat stress. Transgenic *Arabidopsis* plants overexpressing AsHSP26.8a displayed reduced tolerance to heat stress. Furthermore, overexpression of AsHSP26.8a resulted in hypersensitivity to hormone ABA and salinity stress. Global gene expression analysis revealed AsHSP26.8a-modulated expression of heat-shock transcription factor gene, and the involvement of AsHSP26.8a in ABA-dependent and -independent as well as other stress signaling pathways.

**Conclusions:**

Our results suggest that AsHSP26.8a may negatively regulate plant response to various abiotic stresses through modulating ABA and other stress signaling pathways.

## Background

The heat shock proteins (HSPs) are virtually ubiquitous molecules in plants that are rapidly induced by heat stress [1, 2]. At least six types of HSPs, HSP100 (Clp), HSP90, HSP70 (DnaK), HSP60, HSP40 (DNAJ) and small HSPs (sHSPs) have been identified in higher plants, of which the sHSPs with a molecular mass of 12 to 42 kDa are found ubiquitously in all kingdoms of life [3]. The sHSPs function as molecular chaperones, both in vitro and in vivo, to protect cells from stress damage by preventing irreversible protein aggregation and maintaining denatured proteins in a folding-competent state [2, 4–8]. Based on sequence homology, immunological cross-reactivity and subcellular localization, the sHSPs can be further classified into different groups. A total of 12 subfamilies of sHSPs have been identified in various plant species, such as maize (*Zea mays* L.), Kashgar tamarisk (*Tamarix hispida*), creeping bentgrass (*Agrostisc stolonifera* L.), poplars (*Populus*), Pearl millet (*Pennisetum glaucum* L.), tall fescue (*Festuca arundinacea* Schreb.) [9–15]. They are localized in different places within the cell including cytosol or nucleus, mitochondria, plastids (P), endoplasmic reticulum (ER) and peroxisomes (Po) [15–17]. Most of the sHSP subfamily proteins are structurally similar with a highly variable N-terminal region, a conserved core domain of about 100 amino acids and a short C-terminal region [2, 18].

Apart from heat stress, sHSPs are induced in response to a number of other environmental adversities such as osmotic stress (e.g. salt, drought), oxidative stress, cold stress, heavy metal stress and phytohormone ABA [6, 11, 15, 16, 19], suggesting its involvement in plant response to general abiotic stresses. Additionally, their implication in various aspects of plant development have also been documented [16, 20–22].

The roles different sHSPs play in positively regulating plant responses to heat and other environmental stresses (drought, NaCl, mannitol and H_2_O_2_) have largely been reported in various plant species and most of them belong to cytosolic sHSPs [10, 21, 23–32]. Class I and II sHSPs have mainly contributed to the current model for sHSP chaperone activity. These two classes of sHSPs have both unique and overlapping functions and act in conjunction with HSP101 to either directly or indirectly protect specific translation factors in cytosolic stress granules [33]. Extensive evolutionary analysis indicates that organelle-targeted sHSPs (chloroplast, mitochondria, peroxisomes, and endoplasmic reticulum) are closely related to their class I counterparts [34–36]. Endoplasmic reticulum-located sHSP, sHSP22, together with ABA insensitive 1 (ABI1) protein phosphatase controls polar auxin transport and orchestrates ABA and auxin signaling crosstalk in *Arabidopsis* [37]. Peroxisome-located sHSPs activate catalase to regulate plant abiotic stress resistance [38]. A mitochondrial matrix-localized small heat shock protein in cotton, GhHSP24.7, positively controls seed germination via temperature-dependent ROS generation [39]. Overexpression of the chloroplast sHSP has been associated with cold, heat and oxidative stress tolerance [21, 26]. A typical chloroplast-localized sHSP, HSP21, has been identified in many plant species. Several studies have suggested that HSP21 protects the thermolabile photosystem II (PSII) against heat stress [9, 26, 40–42] and oxidative stress [26, 43]. HSP21 is activated by the GUN5-mediated retrograde signaling pathway and stabilizes PSII by directly binding to its subunits under heat stress [44]. HSP21 also interacts with plastid nucleoid protein pTAC5 and is essential for chloroplast development under heat stress by maintaining plastid-encoded RNA polymerase (PEP)-dependent transcription [22]. In addition, HSP21 is involved in extended thermomemory in *Arabidopsis*. Abundant HSP21 during the memory stage is negatively regulated by heat-induced plastid-localized metalloprotease FtsH6 [45]. Our recent study characterizing sHSPs in perennial grasses identified three creeping bentgrass sHSPs, AsHSP17 (previously named ApHSP16.5) (KT272405), AsHSP26.7 (previously named ApHSP26.7) (AY153761) and AsHSP26.8 (previously named ApHSP26.8) (AY153760) [9, 46–48]. *AsHSP17* and *AsHSP26.8* genes were significantly induced in transgenic creeping bentgrass overexpressing a rice SUMO E3 ligase gene, *OsSIZ1* and exhibiting improved heat tolerance compared to wild type controls [46]. In transgenic creeping bentgrass ectopically expressing a cyanobacterial flavodoxin and exhibiting enhanced heat stress tolerance, *AsHSP17*, *AsHSP26.7* and *AsHSP26.8* were all significantly induced but differentially regulated in wild type (WT) and transgenic (TG) plants [47]. Additionally, overexpression of a rice miroRNA, *OsmiR393* improves heat tolerance in transgenic creeping bentgrass that is associated with enhanced expression of sHSP genes, *AsHSP17* and *AsHSP26.7* [48]. Further analysis of AsHSP17, one of these turfgrass sHSPs revealed that AsHSP17 is a negative regulator attenuating plant response to abiotic stress through modulating plant photosynthesis and ABA-dependent and independent signaling [15].

To better understand how sHSPs function in perennial grasses to regulate plant stress response, we focused on *AsHSP26.8*, initially obtained from *A. stolonifera* cv *Penncross* as *ApHSP26.8* [9], and cloned its ortholog gene, *AsHSP26.8a* from *A. stolonifera* cv *Penn-A4* encoding a chloroplast-localized creeping bentgrass sHSP. The cloned *AsHSP26.8a* was introduced into *Arabidopsis* plants for characterization. Transgenic analysis revealed that constitutive expression of *AsHSP26.8a* led to significantly increased plant susceptibility to several abiotic stresses including heat and salt as well as treatment with hormone ABA. Our results suggested that similar to AsHSP17, AsHSP26.8a may also function as a protein chaperone to negatively regulate plant stress response.

## Results

### Cloning and sequence analysis of *AsHSP26.8a* and its protein subcellular localization

In our previous study manipulating sumoylation process in transgenic plants for enhanced tolerance to environmental adversities, it was revealed that overexpression of *OsSIZ1*, a rice E3 SUMO ligase in transgenic creeping bentgrass enhances plant tolerance to a number of abiotic stresses including heat stress associated with altered expression of two sHSP genes, *AsHSP17* and *AsHSP26.8* [46]. Further investigation of AsHSP17 uncovered its role in modulating plant photosynthesis and ABA-dependent and independent signaling to attenuate plant response to abiotic stress including heat and salt stress [15]. We were also curious about how AsHSP26.8 contributes to OsSIZ1-mediated plant stress response and therefore cloned its ortholog gene from the heat-stressed plants of creeping bentgrass cultivar, Penn A-4 and designated it as *AsHSP26.8a*. The *AsHSP26.8a* gene is 729 bp long encoding a protein of 242 amino acids. The predicted polypeptide has a molecular weight (MW) of 26.78 kD and an isoelectric point (pI) of approximately 6.03. Differences in nine nucleotides were identified between *AsHSP26.8a* and *ApHSP26.8* DNA sequences, which led to changes in three amino acids (Fig. S1). Sequence alignment of AsHSP26.8a and the representative plant sHSPs allowed identification of a chloroplast transit peptide (I), a Met-rich region (II) and two consensus regions (III and IV) in AsHSP26.8a (Fig. S2), which was phylogenetically classified as a chloroplast-localized sHSP (Fig. S3).

**Fig. 1:**
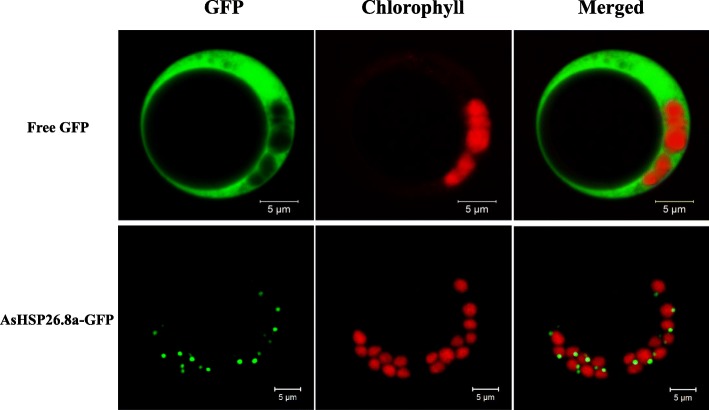
Subcellular localization of AsHSP26.8a protein. Free GFP, control with empty vector; AsHSP26.8a-GFP, AsHSP26.8a-GFP fusion. Bars = 5 μm

To further confirm its subcellular localization in the chloroplasts, AsHSP26.8a-green fluorescent protein (GFP) fusion gene was introduced into rice (*Oryza sativa*) protoplasts. The GFP fluorescence was found to be localized to the nucleoids inside chloroplasts, which is mostly associated with thylakoids (Fig. 1), similar to that of the AtHSP21 (AT4G27670), a well-characterized homolog sHSP of AsHSP26.8a in *Arabidopsis thaliana* [22]. This result indicates that AsHSP26.8a is specifically localized to the thylakoids membranes within chloroplasts.

### *AsHSP26.8a* expression in response to heat stress

**Fig. 2:**
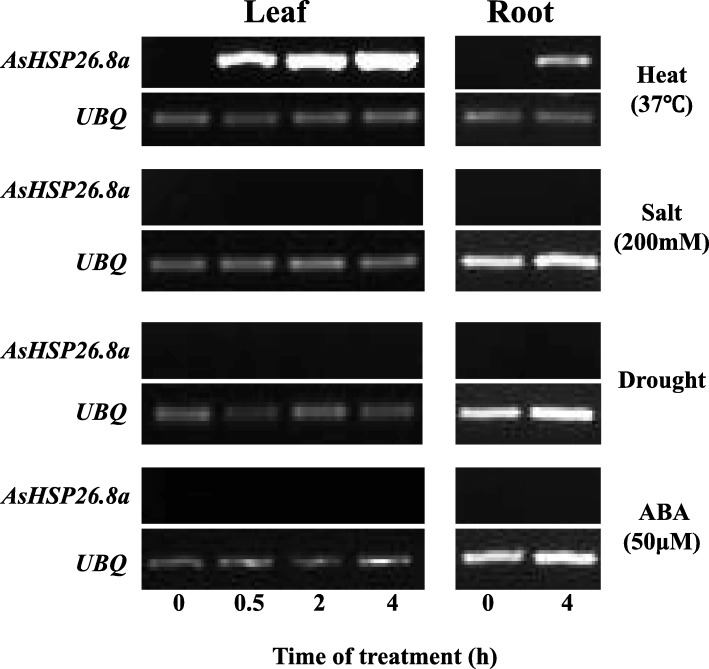
Semi-quantitative RT-PCR analysis of *AsHSP26.8a* expression profile in leaf (left) and root (right) tissues under heat (37 °C), salt (200 mM NaCl), Drought, ABA (50 μM) treatment. Leaf samples were collected at 0, 0.5, 2 and 4 h after stress treatment and root samples were collected at 0 and 4 h after stress treatment. Total RNA was isolated using Trizol and 2 μg total RNA was used for reverse transcription by using reverse transcriptase II (NEB, USA). Creeping bentgrass ubiquitin gene *AsUBQ* was used as the endogenous control. The gel images were cropped to only retain PCR products. All original, full-length gel images were included as additional files in the supplementary materials. The PCR process: 95 °C for 5 min, 30 cycles of 95 °C for 30 s, 65 °C for 30 s, 72 °C for 30 s, for *AsHSP26.8a*; 95 °C for 5 min, 25 cycles of 95 °C for 30 s, 60 °C for 30 s, 72 °C for 30 s, for *AsUBQ*.

Our previous study showed that *AsHSP26.8* is strongly induced by heat stress in creeping bentgrass leaf [46]. To investigate whether the *AsHSP26.8a* responds to heat and other abiotic stresses and ABA stimulus, we examined the expression pattern of the *AsHSP26.8a* in creeping bentgrass under heat, salinity and drought stress as well as exogenous ABA treatment. Total RNAs extracted from leaves and roots were subjected to semi-quantitative RT-PCR analysis. As shown in Fig. 2, the expression of *AsHSP26.8a* in leaves and roots was strongly induced by heat stress, confirming our previous observation [46]. However, no expression was detected in creeping bentgrass leaves or roots subjected to salinity and drought stress as well as ABA treatment (Fig. 2). It should be noted that the very low basal expression level of *AsHSP26.8a* may prevent its detection even if it does respond to salinity, drought and ABA. With the high sequence similarity between *AsHSP26.8a* and other sHSPs such as AsHSP26.2 (AY153578), AsHSP26.7 and AsHSP26.8, we were unable to perform quantitative-real-time reverse transcription PCR (qRT-PCR) analysis for *AsHSP26.8a* expression because of the difficulty in designing appropriate PCR primers.

### Generation of transgenic *A. thaliana* plants constitutively expressing *AsHSP26.8a*

**Fig. 3:**
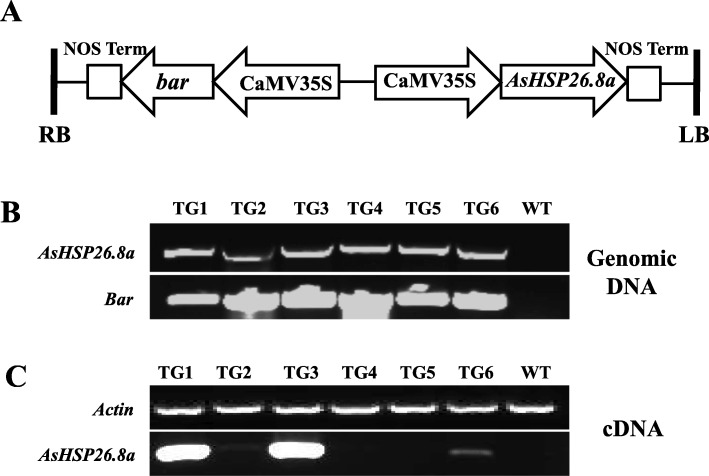
Generation and molecular analysis of the AsHSP26.8a transgenic (TG) *Arabidopsis thaliana.***a,** Schematic diagram of the *AsHSP26.8a* chimeric gene expression construct, p35S-*AsHSP26.8a*/p35S-*bar*. The *AsHSP26.8a* gene and an herbicide resistance gene, *bar*, are both under the control of the CaMV 35S promoter. RB, right border; LB, left border. **b,** PCR analysis of the *AsHSP26.8a* and *bar* gene in wild type (WT) and TG plants to detect transgene insertion in the *Arabidopsis thaliana* genome and transcription. Actin gene *AtActin1* was used as the endogenous control. The gel images were cropped to only retain PCR products. All original, full-length gel images were included as additional files in the supplementary materials

To further investigate the role of AsHSP26.8a in plant response to abiotic stress, we set to manipulate *AsHSP26.8a* expression in transgenic plants. To this end, an *AsHSP26.8a* overexpression vector was constructed (Fig. 3a) for transformation into *A. thaliana* ecotype Columbia. The six independent transgenic lines generated (Fig. 3b, c) did not appear different from wild type controls, of which four homozygous lines, TG1, TG2, TG3 and TG6 expressing different levels of *AsHSP26.8a* (Fig. 3b, c) were further analyzed. It should be noted that in this study, we chose to use wild type plants as the control in characterizing AsHSP26.8a transgenic plants based on the observation that their development and response to various stresses exhibited no significant difference from that of the transgenic lines harboring an empty vector or a *gus* reporter gene (data not shown).

### *AsHSP26.8a* overexpression leads to increased heat susceptibility in transgenic plants

**Fig. 4:**
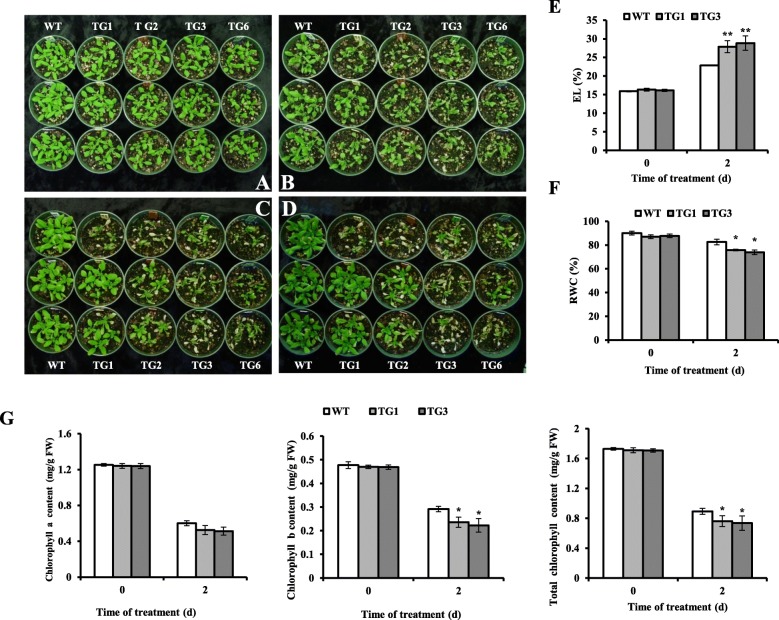
Responses of wild type (WT) and transgenic (TG) plants to heat stress. Two-week-old *Arabidopsis thaliana* WT and four AsHSP26.8a TG lines were grown under normal conditions in a growth chamber (**a).** Heat stress was applied by heating the plants to 40 °C for 2 d (**b).** The plants were then moved back to normal conditions, photographed 2 and 4 d after recovery (**c** and **d**). Leaf samples collected 2 d after heat stress were used for measuring electrolyte leakage (**e**), relative water content (**f**) and chlorophyll a content (**G** left), Chlorophyll b content (**G** middle) and total chlorophyll content (**G** right) (*n* = 3). Each column represents mean of three biological replicates. Error bars represent SE. ‘*’ or ‘**’ indicate significant differences between TG and WT plants at *P* < 0.05 or 0.01, respectively by Student’s t test

To evaluate how altered *AsHSP26.8a* expression impacts plant response to high temperature, we subject the well-developed 3 weeks old AsHSP26.8a-overexpressing transgenic plants to heat stress (40 °C) and evaluated their performance in comparison with non-transgenic plants. As shown in Fig. 4b, two-day heat stress led to non-recoverable severe damage in the majority of the transgenic plants, whereas the heat-elicited damage was barely observed in wild type controls, all of which recovered and survived the treatment (Fig. 4c, d). Plant relative water content (RWC) and electrolyte leakage (EL) were measured to exam their water status and cell membrane integrity. While similar RWC and EL were observed in both transgenic and wild type control plants under normal growth conditions, there was a significantly more water loss and a more severe heat-elicited cell membrane damage in the AsHSP26.8a-expressing transgenics than in the control plants 2 days after heat treatment (Fig. 4e, f), suggesting that AsHSP26.8a negatively impacts plant water retention capacity and cell membrane integrity. Similarly, although no significant difference in chlorophyll a, b and total chlorophyll contents was observed between wild type control and the AsHSP26.8a-containing plants under normal growth conditions, the AsHSP26.8a-expressing transgenic plants exhibited significantly lower leaf chlorophyll a, b and total chlorophyll contents than wild type control under heat-stress conditions (Fig. 4g) suggesting a reduced chlorophyll production in AsHSP26.8a transgenic plants under heat stress. It should be noted that the inconsistent phenotypes of the TG1 plants in the three pots used for heat stress response assessment (Fig. 4a-d) was most likely due to random experimental errors rather than the inherent difference between individual transgenic plants.

### AsHSP26.8a transgenic plants exhibit increased salinity susceptibility

**Fig. 5:**
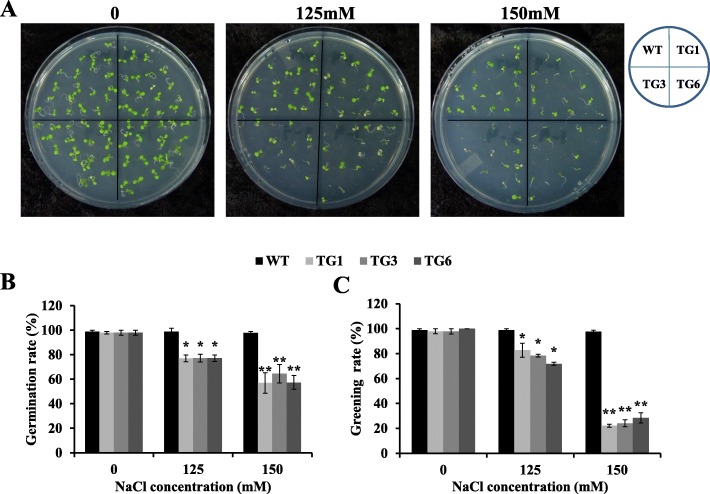
Germination and greening rates of wild type (WT) and transgenic (TG1, TG3 and TG6) plants in response to salt treatment. **a**, seed germination of WT and AsHSP26.8a TG plants subjected to 0, 125 and 150 mM of NaCl 6 d after treatment. **b,** germination and **c,** greening percentages of WT and TG seeds subjected to 0, 125 and 150 mM NaCl 6 d after treatment (*n* = 3). Each column represents mean of three biological replicates. Error bars represent SE. ‘*’ or ‘**’ indicate significant differences between TG and WT plants at P < 0.05 or 0.01 respectively by Student’s t test. The layout of the plates is shown on the upper right-hand corner

To investigate possible involvement of AsHSP26.8a in plant response to other abiotic stresses, we examined seed germination of AsHSP26.8a overexpressing plants subjected to salt stress (Fig. 5) and observed that transgenic plants exhibited significantly lower seed germination than the wild type controls when treated with 125 or 150 mM NaCl, under which the germination rate was only approximately 60–80% in the AsHSP26.8a transgenics versus 100% in wild type controls (Fig. 5a, b). Similarly, plant post-germination growth in *AsHSP26.8a*-expressing transgenic plants was also significantly impaired compared to wild type controls under the two different NaCl concentrations (Fig. 5a, c). These data indicate that besides heat stress, AsHSP26.8a also impacts how plants tackle other environmental adversities. More specifically, it negatively regulates plant salinity stress response.

### *AsHSP26.8a* overexpression impacts ABA signaling in transgenic plants

**Fig. 6:**
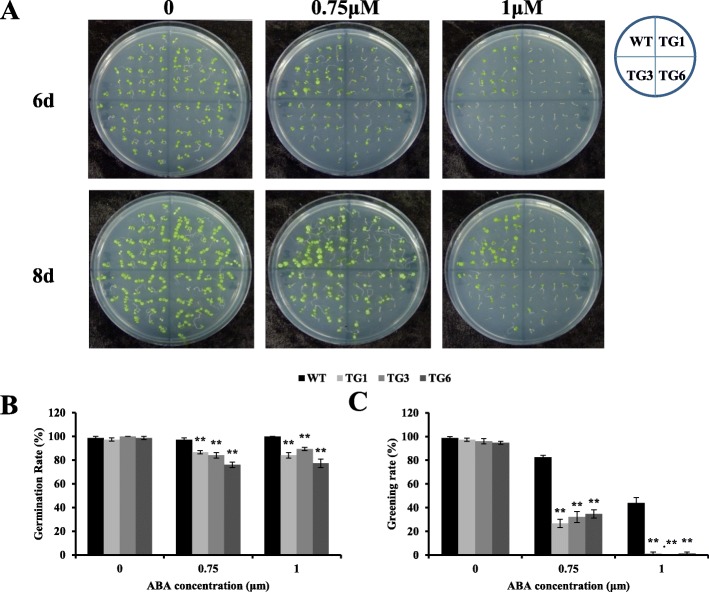
Germination and greening rates of wild type (WT) and transgenic (TG1, TG3 and TG6) plants in response to abscisic acid (ABA). **a**, seed germination of WT and AsHSP26.8a TG plants subjected to 0, 0.75 and 1 μM of ABA 6 and 8 d after treatment. **b**, germination percentages of WT and TG seeds subjected to 0, 0.75 and 1 μM of ABA 6 d after treatment (*n* = 3). **C**, greening percentages of WT and TG seeds (percentages of seeds with fully expanded cotyledons) subjected to 0, 0.75 and 1 μM of ABA 6 d after treatment (*n* = 3). Each column represents mean of three biological replicates. Error bars represent SE. ‘*’ or ‘**’ indicate significant differences between TG and WT plants at P < 0.05 or 0.01 respectively by Student’s t test. The layout of the plates is shown on the upper right-hand corner

Among various phytohormones, ABA is the central regulator of abiotic stress resistance in plants and coordinates an array of functions [49–51]. We were curious about the possible involvement of AsHSP26.8a in plant ABA signaling. To this end, we conducted experiments assessing plant response to exogenous ABA treatment (0, 0.75 and 1 μM) and found that compared to wild type controls, seed germination and post germinative plant growth in AsHSP26.8a transgenics were both significantly reduced responding to ABA application (Fig. 6), suggesting that AsHSP26.8a may function as a positive regulator mediating ABA-associated plant development.

### Genome-wide gene expression analysis in AsHSP26.8a overexpression transgenic plants

**Fig. 7:**
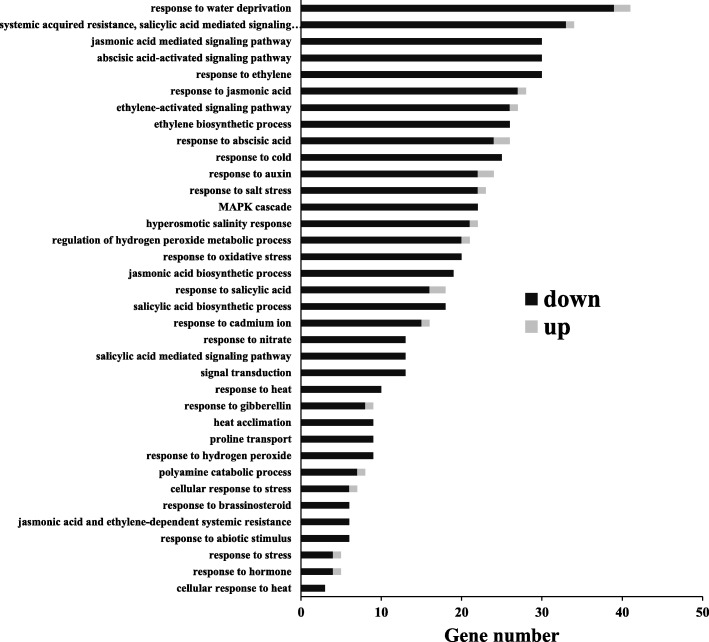
Gene ontology (GO) classification for abiotic stress-related differentially expressed genes (DEGs) in wild type (WT) and AsHSP26.8a transgenic (TG) plants. The y-axis and x-axis indicate the names of clusters and the gene number of each cluster, respectively. Only the biological processes were used for GO term analysis

To better understand molecular mechanisms underlying AsHSP26.8a involvement in plant abiotic stress response, we conducted genome-wide gene expression analysis in wild type and AsHSP26.8a transgenic plants to screen for differentially expressed genes (DEGs) between the two genotypes (Table S1). Among a total of 269 DEGs identified, 20 (7%) were up-regulated and 249 (93%) down-regulated. Absolute log2 fold changes ranged from − 6.80 to 4.84 and adjusted *P*-value (FDR) ranged from 1.61 × 10^− 77^ to 0.99 × 10^− 2^. Allocated gene ontology (GO) term-based classification of the 269 DEGs resulted in 53 different groups. One of the most dominant GO terms was ‘Response to stimulus’ in the biological process category (Fig. S4). Additionally, many genes responsive to phytohormones (ABA, ethylene, jasmonic acid, auxin, salicylic acid, gibberellin) and abiotic stresses (drought, cold, salinity, oxidative stress, cadmium ion and heat) as well as those involved in various stress-related signaling pathways (salicylic acid mediated signaling pathway, jasmonic acid mediated signaling pathway, abscisic acid-activated signaling pathway, ethylene-activated signaling pathway) were also identified (Fig. 7).

A total of 88 abiotic stress-related DEGs identified from the RNA-seq data were all functionally annotated that encode either regulatory or functional proteins (Table 1). The regulatory proteins consist of 30 transcription factors (e.g. AP2/ERF, DREB, HSF, MYB, NAC, WRKY and Zinc finger protein), 10 signaling proteins (e.g. calcium-binding proteins) and 17 kinases (e.g. cysteine-rich receptor-like protein kinase, leucine-rich repeat protein kinase, mitogen-activated protein kinase kinase kinase 14, S-locus lectin protein kinase family protein and wall-associated receptor kinase-like 2). The functional proteins include six cytochrome P450 family members, three LEA proteins, four carbohydrate metabolism-related proteins, thirteen nitrogen metabolism-related proteins, four proteins involved in oxidation-reduction process and one AAA-type ATPase family protein. The majority of these 88 abiotic stress-related DEGs were down-regulated except six encoding one MYB domain protein, one protein kinase, one cytochrome P450 family member, one alpha/beta-hydrolase family protein and two proteins involved in oxidation-reduction process). The differential expression patterns of the four representative abiotic stress responsive genes, DREB1B, ERF105, HSFB2a and HSFC1 between wild type control and the AsHSP26.8a transgenic plants were confirmed by qRT-PCR analysis (Table 2), further validating the RNA-seq data.

**Table 1:** Annotation of genes up-regulated or down-regulated in AsHSP26.8a overexpressing transgenic (TG) *Arabidopsis* compared to wild type (WT) controls. Significant differences were corrected with FDR < 0.01 and expression ratio ≥ 2, (FC, fold change)

Gene #ID	log2FC	nr_annotation
**Signal proteins**		
**Transcription factor**		
AT1G68840	−1.12	AP2-EREBP family, RAVE subfamily protein RAV2
AT4G25490	−6.80	dehydration-responsive element-binding protein 1B
AT3G15210	−2.12	ethylene-responsive transcription factor 4
AT5G47230	−2.49	ethylene-responsive transcription factor 5
AT5G53290	−2.27	ethylene-responsive transcription factor CRF3
AT3G50260	−2.03	ethylene-responsive transcription factor ERF011
AT1G77640	−3.41	ethylene-responsive transcription factor ERF013
AT1G22190	−1.81	ethylene-responsive transcription factor ERF058
AT5G61600	−2.52	ethylene-responsive transcription factor ERF104
AT5G51190	−4.49	ethylene-responsive transcription factor ERF105
AT5G62020	−1.50	heat stress transcription factor B-2a
AT3G24520	−1.68	heat stress transcription factor C-1
AT5G49330	1.39	myb domain protein 111
AT1G18570	−1.31	myb domain protein 51
AT4G37260	−1.35	myb domain protein 73
AT3G50060	−2.70	myb domain protein 77
AT1G25550	−1.63	myb-like transcription factor-like protein
AT5G67300	−1.39	transcription factor MYB44
AT1G69490	−1.26	NAC transcription factor protein family
AT4G31800	−1.18	WRKY DNA-binding protein 18
AT1G62300	−1.16	WRKY transcription factor 6
AT3G46620	−3.92	C3H4 type zinc finger protein
AT1G51700	−1.19	DOF zinc finger protein 1
AT5G59820	−4.87	high light responsive zinc finger protein ZAT12
AT3G52800	−1.56	zinc finger A20 and AN1 domain-containing stress-associated protein 6
AT1G27730	−3.74	zinc finger protein STZ/ZAT10
AT2G37430	−5.33	zinc finger protein ZAT11
AT5G04340	−2.28	zinc finger protein ZAT6
AT3G46090	−4.18	zinc finger protein ZAT7
AT5G67450	−3.06	zinc-finger protein 1
**Signaling**		
AT5G49480	−1.41	Ca2 + −binding protein 1
AT5G37770	−2.55	calcium-binding protein CML24
AT1G76650	−4.92	calcium-binding protein CML38
AT5G62570	−1.17	calmodulin binding protein-like protein
AT1G66400	−3.17	calmodulin like 23
AT2G41100	−1.02	calmodulin-like protein 4
AT3G25600	−1.50	putative calcium-binding protein CML16
AT3G29000	−4.55	putative calcium-binding protein CML30
AT5G39670	−2.02	putative calcium-binding protein CML45
AT3G10300	−1.02	putative calcium-binding protein CML49
**Kinase**		
AT4G23190	−1.43	cysteine-rich receptor-like protein kinase 11
AT2G19190	−2.16	FLG22-induced receptor-like kinase 1
AT1G67470	−1.37	inactive serine/threonine-protein kinase
AT5G01540	−1.60	lectin receptor kinase A4.1
AT1G33610	−1.28	leucine-rich repeat (LRR) family protein
AT1G51790	−1.02	leucine-rich repeat protein kinase-like protein
AT3G47090	−1.02	leucine-rich repeat protein kinase-like protein
AT2G30040	−2.38	mitogen-activated protein kinase kinase kinase 14
AT1G51890	−1.73	probable LRR receptor-like protein kinase
AT2G44830	1.01	protein kinase
AT3G57640	−1.24	protein kinase family protein
AT4G11521	−1.26	putative cysteine-rich receptor-like protein kinase 34
AT4G04540	−2.83	putative cysteine-rich receptor-like protein kinase 39
AT1G51800	−1.51	putative leucine-rich repeat protein kinase
AT1G74360	−1.12	putative LRR receptor-like serine/threonine-protein kinase
AT1G61370	−1.21	S-locus lectin protein kinase family protein
AT1G16130	−1.09	wall-associated receptor kinase-like 2
**Function protein**		
**Cytochrome P450**		
ATCG00730	−1.68	cytochrome b6/f complex subunit IV
AT4G31500	−1.07	cytochrome P450 83B1
AT4G22710	−2.44	cytochrome P450, family 706, subfamily A, polypeptide 2
AT4G12320	1.32	cytochrome P450, family 706, subfamily A, polypeptide 6
AT5G57220	−1.43	cytochrome P450, family 81, subfamily F, polypeptide 2
AT2G27690	−1.56	cytochrome P450, family 94, subfamily C, polypeptide 1
**LEA**		
AT1G65690	−1.51	late embryogenesis abundant (LEA) hydroxyproline-rich glycoprotein
AT4G23610	−2.52	late embryogenesis abundant hydroxyproline-rich glycoprotein
AT3G54200	−1.07	late embryogenesis abundant hydroxyproline-rich glycoprotein
**Carbohydrate metabolism-related proteins**		
AT3G09020	−1.37	alpha 1,4-glycosyltransferase family protein
AT5G26340	−1.37	sugar transport protein 13
AT5G18840	−2.66	sugar transporter ERD6-like 16
AT3G49790	−1.18	Carbohydrate-binding protein
**Nitrogen metabolism-related proteins**		
AT1G02920	−1.26	glutathione S-transferase 7/11
AT2G02930	−1.48	glutathione S-transferase F3
AT5G62480	−2.05	glutathione S-transferase tau 9
AT5G02780	−1.78	glutathione transferase lambda 1
AT1G77760	−1.38	nitrate reductase [NADH]
AT1G66180	−1.61	aspartyl protease family protein
AT5G19120	−1.14	aspartyl protease family protein
AT2G38860	−1.03	protease I (pfpI)-like protein YLS5
AT4G22470	−1.24	protease inhibitor/seed storage/lipid transfer protein (LTP) family protein
AT4G14290	4.42	alpha/beta-hydrolase family protein
AT1G02660	−1.37	alpha/beta-Hydrolases superfamily protein
AT3G05200	−1.06	E3 ubiquitin-protein ligase ATL6
AT3G52450	−2.75	E3 ubiquitin-protein ligase PUB22
**Oxidation-reduction process**		
AT3G09940	−1.38	monodehydroascorbate reductase (NADH)
AT4G37925	1.01	NAD(P)H-quinone oxidoreductase subunit M
AT4G11290	−1.27	peroxidase 39
AT3G47430	1.25	peroxisomal membrane protein 11B
**Energy**		
AT3G28510	−2.42	AAA-type ATPase family protein

**Table 2:** Differentially expressed genes in AsHSP26.8a overexpression transgenic (TG) and wild type (WT) *Arabidopsis* plants by qRT-PCR analysis

Gene	TG/WT (log2 FC)	
	RNA-seq	qRT-PCR
*DREB1B*	−6.80	−6.35
*ERF105*	−4.49	−4.82
*HSFB2a*	−1.68	−2.53
*HSFC1*	−1.50	−2.17

## Discussion

The sHSPs, especially those in plants, are a large and complex family of proteins [35]. The plant sHSPs are divided into 12 subfamilies, of which chloroplast-localized sHSPs family has been identified in diverse higher plant species [52, 53]. This family of sHSPs have a Met-rich domain and a unique amphipathic domain at their N-terminus, not present in other sHSPs. AsHSP26.8a possesses both a Met-rich domain and a chloroplast transit peptide at its N-terminus (Fig. S2). Phylogenetic analysis and subcellular localization further support the classification of the AsHSP26.8a protein as a member of the chloroplast-localized sHSPs family (Fig. S3 and Fig. 1).

### AsHSP26.8a-mediated HSP/HSF pathway and plant abiotic stress tolerance

The major abiotic stresses such as drought, high salinity, extreme temperature, negatively influence plant survival, growth and productivity. As sessile organisms, plants are unable to change their sites to escape from the unfavorable environmental adversities but have developed a great degree of resilience to conditions that would be considered harmful to many other organisms. A network of interconnected cellular stress response systems is essential for plant survival and productivity [54]. Within the complex stress response network, transcription factors (TFs) play a core role in the conversion of stress signal perception to stress-responsive gene expression by interacting with the promoter regions of various target stress-responsive genes, thus activating the whole network of genes to act together in enhancing plant tolerance to the harsh environmental conditions [55]. Heat shock transcription factors (HSFs) are the central regulators in plant cellular response to various abiotic stresses, especially to heat stress [56, 57]. Class B HSF and Class C HSF have been implicated in plant response to heat stress. HSFB1 and HSFB2b repress the expression of HSFs, but positively impact the acquired thermotolerance [58]. *Capsicum annuum* HSFB2a forms a transcriptional cascade with CaWRKY6 and CaWRKY40 to positively regulate the response to high temperature and high humidity [59]. Guan et al. [60] found that regulation of heat stress-responsive genes including HSFC1 and other HSFs by RCF2 and its interacting partner NAC transcription factor NAC019 is critical for thermotolerance in *Arabidopsis*. A recent study showed that HSFC1b from tall fescue plays a positive role in plant tolerance to heat stress in association with the induction and upregulation of heat-protective genes [61]. Our results showed that overexpression of *AsHSP26.8a* alters plant heat stress response (Fig. 4) and results in down-regulated expression of the two HSFs, HSFB2a and HSFC1 in transgenic plants (Table 1), suggesting that AsHSP26.8a may function to repress HSF gene expression, modulating heat-responsive genes and thus attenuating plant response to heat stress.

### AsHSP26.8a modulates ABA-dependent stress signaling and plant abiotic stress response

ABA, commonly known as the “stress hormone”, responds to an array of biotic and abiotic stresses [62]. Under osmotic stress condition such as drought and high salinity stress, a number of genes functioning in stress response and tolerance are induced, and ABA is accumulated [49, 63, 64]. The expression of stress-responsive genes is regulated by ABA-dependent and ABA-independent pathways [64]. These genes encode the late embryogenesis abundant (LEA) proteins, enzymes, transcription factors, protein kinases et al. LEA accumulation is a functional adaptation of plants in gaining tolerance against osmotic as well as oxidative stresses [65]. Overexpression of genes encoding LEA proteins can improve the stress tolerance of transgenic plants [66–69]. MYB transcription factors are a large group of proteins identified in eukaryotes and widely distributed in plants [70–72]. Some of the MYB protein family members are involved in ABA-dependent signaling pathways regulating stress adaption and conferring plant stress tolerance [71, 73–75]. In this study, overexpression of *AsHSP26.8a* alters plant development and plant response to ABA and salt stress (Figs. 5 and 6) and leads to significantly reduced expression of several genes encoding LEA and MYB proteins (Table 1) as well as some stress-responsive transcription factors, such as WRKY transcription factors, involved in ABA-dependent signaling [76]. Abiotic stresses such as heat, high salinity and drought also induce the WRKY genes and trigger a cascade of signaling pathways for improved plant stress tolerance [77, 78]. Many studies showed that overexpression of a WRKY family gene confers abiotic stress tolerance in transgenic plants. For example, AtWRKY25 and AtWRKY26 overexpression enhanced plant heat tolerance in transgenic *Arabidopsis* [79]. Transgenic *Arabidopsis* overexpressing a wheat WRKY transcription factor, TaWRKY33 exhibited enhanced heat tolerance [80]. In cotton, GhWRKY17 overexpression increased plant sensitivity to drought and salt stress as well as ABA-mediated seed germination and root growth by reducing the levels of ABA and transcripts of ABA-inducible genes including *NbAREB1* (ABA-responsive element binding), *NbDREB* (dehydration-responsive element binding), *NbNCED* (9-cis-epoxycarotenoid dioxygenase), *NbERD* (early responsive to dehydration) and LEA protein, *NbLEA* [81]. These data suggest that AsHSP26.8a may function as a chaperone protein, contributing to ABA-dependent signaling in plant abiotic stress responses.

### AsHSP26.8a modulates ABA-independent stress signaling and plant abiotic stress response

Our results also showed that AsHSP26.8a modulates stress-related transcription factor gene expression in the ABA-independent signaling pathways. The ABA-independent stress-responsive gene expression is regulated by DREB proteins. DREBs belong to Ethylene Response Factor (ERF)/AP2 family and consist of two subclasses, DREB1/CBF and DREB2 induced by cold and dehydration/high salinity, respectively [64, 82–89]. *Arabidopsis* DREB1A overexpression was reported to enhance LEA protein levels and therefore abiotic stress tolerance in *Arabidopsis* [90, 91] and various crops including rice, soybean, peanut and wheat [92–95]. Heterologous expression of AtDREB1B in *Salvia miltiorrhiza* enhanced plant drought tolerance by activating different downstream DREB/CBF genes [96]. Moreover, AtHSFA3 is a transcription factor that is transcriptionally induced during heat stress by DREB2A and in turn regulates the expression of HSP-encoding genes [97]. Overexpressing AtDREB2A in *Arabidopsis* plants induces not only drought- and salt-responsive genes but also heat-shock-related genes. Thermotolerance was significantly increased in plants overexpressing DREB2A and decreased in DREB2A knockout plants [85]. The ERF (ethylene-responsive element binding factor) is another subfamily of the AP2/ERF family of TFs and plays vital roles in the regulation of biotic and abiotic stress responses [98–100]. Overexpression of a tomato ERF transcription factor, SlERF84 in *Arabidopsis* endows transgenic plants with ABA hypersensitivity and enhanced tolerance to drought and salt stress [101]. Overexpression of CmERF053 of *chrysanthemum* could enhance drought tolerance [102]. In *Tamarix hispida*, constitutive expression of an ERF transcription factor, ThCRF1, increased biosynthesis of trehalose and proline and the activities of SOD and POD, resulting in an altered osmotic potential and an enhanced reactive oxygen species (ROS) scavenging, and therefore significantly improved salt tolerance in transgenic plants. On the contrary, suppression of ThCRF1 led to decreased plant salt tolerance [103]. In this study, transgenic plants overexpressing AsHSP26.8a displayed significant expression changes in ten ERF/AP2 family genes (Table 1), for example, compared to the wild type, *DREB1B/CBF1* and *ERF105* expression in AsHSP26.8a transgenic plants was down-regulated over sixty-fold and thirty-fold, respectively (Table 1). These results suggest that AsHSP26.8a-modulated expression of the genes in the ABA-independent signaling pathways may also contribute to plant response to various abiotic stresses.

### Other stress signaling pathways mediated by AsHSP26.8a and plant abiotic stress response

Calcium (Ca^2+^) is the most widely accepted second massager and involved in plant stress responses and cytoplasmic Ca^2+^ signal is recognized by Ca^2+^ sensors including calmodulins (CaM), calmodulin-like proteins (CMLs), calcium dependent protein kinases (CDPKs) and calcineurin B-like proteins (CBLs) [104–111]. Overexpression of AtCML24 enhances transgenic *Arabidopsis* tolerance to various ions including Co^2+^, Zn^2+^ and Mg^2+^ [112]. CML18 directly interacts with Na^+^/H^+^ antiporter NHX1 to regulate plant salinity tolerance [113]. CML9 is suggested to negatively regulate ABA-dependent salinity tolerance [114]. OsANN1, a calcium-binding protein of rice modulates antioxidant accumulation under abiotic stress to confer abiotic stress tolerance. OsANN1-knockdown led to increased plant sensitivity to heat and drought stresses, whereas OsANN1 overexpression resulted in improved plant growth with higher expression of OsANN1 under abiotic stress [115]. Receptor-like protein kinases (RLKs), a class of single-pass transmembrane proteins located in the plasma membrane, sense and transmit a variety of signals to regulate plant growth and development [116, 117]. Many RLKs have been implicated in abiotic stress responses, including the abscisic acid response, calcium signaling and antioxidant defense. Upon drought stress, the *Arabidopsis* LRK10L1.2 responds to directly or indirectly regulate stomata closure via ABA-mediated signaling [118], while a *Glycine soja* ABA-responsive receptor-like cytoplasmic kinase (RLCK), GsRLCK, responds to modulate ABA sensitivity in plants by regulating the expression of ABA-responsive genes [119]. In *Pisum sativum*, salinity induced lectin receptor-like kinase (*PsLecRLK*) gene expression and overexpression of *PsLecRLKs* led to improved plants salt tolerance due to enhanced ROS-scavenging [120]. In *Medicago* spp., the LRR-RLK gene, SRLK has been shown to regulate the root response to salt stress [121]. Large families of zinc finger transcription factors are abundant in plants and have diverse functions including DNA binding and transcriptional regulation [122]. Cys2/His2-type (C2H2) zinc finger proteins are implicated in plant response to a variety of adversities including low-temperatures, salt, drought, oxidative stress, excessive light and silique shattering [123–125]. One example is ZAT6 in *Arabidopsis* that positively regulates cadmium tolerance via the glutathione-dependent pathway [126]. Another example is the C2H2 zinc finger protein gene, *Zat7* whose constitutive expression suppressed growth and enhanced salt tolerance in transgenic *Arabidopsis* plants [127]. Moreover, transgenic analysis in *Arabidopsis* points to the involvement of ZAT10 or STZ (salt tolerance zinc finger) in determining plant tolerance to drought, salt, osmotic, heat, photo-inhibitory light and oxidative stresses [128–131]. In this study, AsHSP26.8a overexpression led to significantly reduced expression of the genes encoding four CMLs, fifteen RLKs and nine zinc finger proteins in transgenic *Arabidopsis* (Table 1). For example, *ZAT11* expression in AsHSP26.8a TG *Arabidopsis* plants was down-regulated over thirty-fold and *ZAT12*, *ZAT7*, *CML38* and *CML30* were down-regulated over sixteen-fold compared to wild type controls (Table 1). These results suggest that other than the ABA-dependent and -independent signaling pathways, AsHSP26.8a may also participate in other signaling pathways responding to abiotic stresses.

A few studies about the negative effect of HSPs on plant response to abiotic stress have previously been reported. Song et al. [132] found that transgenic *Arabidopsis* overexpressing cytosolic and organellar AtHSP90s exhibited suppressed expression of stress-responsive genes and consequently reduced salt and drought tolerance. Our previous study also showed that overexpression of AsHSP17, a creeping bentgrass sHSP, attenuates plant response to abiotic stress by modulating plant photosynthesis and ABA-dependent and -independent signaling [15]. Moreover, Wang and Luthe [9] were unable to amplify *ApHSP26.8* (renamed as *AsHSP26.8* in this study) from the heat-tolerant variant selected bentgrass, which was regenerated from callus that survived selection at 40 °C for 1 week, but they were able to amplify the gene from the heat-sensitive variant non-selected bentgrass, which was not subjected to heat stress. Taken together, these data imply that similar to AsHSP17, as a chaperone protein, AsHSP26.8a may be regulated to maintain an appropriate level in protecting a stressed plant. The excessive amount of AsHSP26.8a in transgenic plants may negatively impact the stress response regulatory network, compromising on plant stress tolerance. Indeed, our results assessing plant performance under stressful conditions showed that the plant response to heat stress in different transgenic lines appeared quite consistent regardless of the level of the elevated *AsHSP26.8a* expression (Fig. 3c and Fig. 4b-d). It is therefore tempting to speculate that any change in *AsHSP26.8a* expression above the basal level would have significant impact on plant response to environmental stress. Alternatively, AsHSP26.8a may need to stay inactive under normal condition not to become a stress itself. Using transgenic approach, we are currently investigating the impact of the regulated expression of *AsHSP26.8a* in creeping bentgrass itself in order to better understand molecular mechanisms of AsHSP26.8a-mediated plant development and stress response.

## Conclusions

We have cloned and characterized a chloroplast localized sHSP gene, *AsHSP26.8a*, whose expression is induced by heat stress. Overexpression of *AsHSP26.8a* in transgenics attenuates plant response to various abiotic stresses including heat, salt and ABA. AsHSP26.8a may be involved in several aspects of plant stress response including ABA-dependent and -independent signaling and some other stress response pathways. Although the molecular mechanisms of the possible chaperone role AsHSP26.8a may play in plant stress response remain to be unraveled, the results obtained from the current study allow a better understanding of sHSPs involvement in plant abiotic stress response providing information for prospecting studies towards the development of novel molecular strategies for enhancing crop performance under adverse environments.

## Methods

### Plant materials and abiotic stress treatment

The creeping bentgrass cultivar ‘Penn-A4’ originally provided by HybriGene (Hubbard, OR) was clonally propagated from stolon and maintained as described previously [133]. Plants were maintained in the growth room and subjected to heat, salt, drought and phytohormone treatment as described previously [15]. The leaf samples were collected at 0, 0.5, 2 and 4 h and roots were collected at 0 and 4 h after stress treatment for RNA extraction to clone the *AsHSP26.8a* gene and analyze *AsHSP26.8a* gene expression.

The wild type *A. thaliana* plants (ecotype Columbia) and four transgenic lines (TG1, TG2, TG3 and TG6) generated by Xinbo Sun were cultured in a growth chamber and subjected to heat stress as described previously [15]. The seeds of the transgenic lines obtained are maintained and available in Hong Luo’s lab at Clemson University (Clemson, SC).

### Plant genomic DNA, RNA isolation and gene expression analysis

Plant genomic DNA was extracted as described previously [134]. Plant total RNA was extracted from 100 mg of fresh leaves/roots with Trizol reagent (Invitrogen, Carlsbad, CA) following the manufacturers’ protocol. First-stand cDNA was synthesized from 2 μg RNA with SuperScript III System (Invitrogen) and oligo (dT) or gene specific primers.

Semi-quantitative RT-PCR was conducted on 25–30 cycles based on its exponential phase. PCR products were analyzed by electrophoresis using a 0.8% or 1.5% (w/v) agarose gel and photographed with the BioDoc-It imaging system (Ultra -Violet Products). The creeping bentgrass ubiquitin gene, *AsUBQ* (JX570760) and *A. thaliana Actin1* (AT2G37620) were used as reference genes.

Using the Bio-Rad iQ5 real-time detection system with 12.5 μL of iQ SYBR Green Supermix (Bio-Rad Laboratories, Hercules, CA), quantitative real-time PCR was conducted to verify the expression of four representative DEGs (*DREB1B, ERF105, HSFB2a* and *HSFC1*) in WT and TG *Arabidopsis* plants identified by RNA-seq analysis. The reaction mix was preincubated at 95 °C for 3 min followed by 40 cycles of denaturing at 95 °C for 30 s, annealing at 60 °C for 30 s, extension at 72 °C for 20 s. The data were collected by using iQ5 Optical System Software version 2.0 (Bio-Rad Laboratories) with *AtActin1* and *AtTub6* (AT5G12250), the two reference genes as endogenous controls for *A. thaliana* analysis. The ΔΔCt method was used for real-time PCR analysis with three biological replicates [133].

### Subcellular localization of GFP protein in rice protoplasts

For subcellular localization, the full-length of *AsHSP26.8a* without a stop codon was subcloned into the pUC19/35S-EGFP vector with GFP at C terminus. The resulting fusion construct and empty vector were transformed into rice protoplasts by PEG (polyethylene glycol)-mediated transformation method, respectively [135]. After incubation in the dark for 16 h, the GFP fluorescence of transiently transformed rice protoplasts was examined and photographed under a confocal scanning microscopy (LSM510 Meta; Zeiss). For GFP, we used 488 and 519 nm for excitation and emission, respectively. For chlorophyll autofluorescence, we used 488 and 650–750 nm for excitation and emission, respectively.

### Plasmid construction and plant transformation

The *AsHSP26.8a* overexpression construct, p35S-*AsHSP26.8a*/35S-*bar* contains the open reading frame of the turfgrass sHSP gene, *AsHSP26.8a*, driven by the cauliflower mosaic virus 35S (CaMV35S) promoter and linked to a CaMV35S-driven *bar* gene for herbicide resistance. The construct was introduced into wild type *A. thaliana* (Col-0) by *Agrobaterium tumefaciens* (LBA4404)-mediated plant transformation using floral dip. Individual transgenic plants were selected by herbicide screening.

### RNA-seq analysis

The 4-week-old wild type and AsHSP26.8a TG3 seedlings were harvested for total RNA isolation with Trizol reagent (Invitrogen, Carlsbad, CA). RNA concentration, purity and integrity were monitored by Nanodrop, Qubit 2.0 and Agilent 2100 Bioanalyzer. The mRNAs were isolated using oligo (dT) magnetic beads and randomly broken into 150- to 250- pieces in fragmentation reagent. The first strand cDNA was generated by employing random hexamer-primers and reverse transcriptase, followed by the synthesis of the second-strand cDNA in the presence of dNTPs, RNase H and DNA polymerase I. The purified cDNA products after end-reparation, adaptor ligation and size selection by AMPure XP beads were PCR-amplified and then sequenced on an Illumina HiSeq2500 (Biomaker Technologies, Beijing, China). The clean reads obtained after removal of low-quality reads and adaptor sequences from the raw reads of each library were mapped to the reference *Arabidopsis* genome (TAIR 10, ftp://ftp.ensemblgenomes.org/pub/ plants/release-25/fasta/arabidopsis_thaliana/) using TopHat2 [136]. The FPKM (fragments per kilobase of transcript per million fragments mapped) values were used to measure Gene expression levels [137]. The FDR (false discovery rate) values of ≤0.01 and the FC (fold change) value of ≥2 (the absolute value of log2 ratio ≥ 1) were used to determine differentially expressed genes (DEGs) in the four libraries from wild type and AsHSP26.8a TG plants (two libraries from two biological replicates for each sample). Gene Ontology (GO) enrichment analysis was conducted by mapping all the DEGs to GO terms in the GO database (http://www.geneontology.org/) and the unigene number in each term was also determined.

Pearson’s correlation coefficients [138] between each pair of the biological replicates in WT and TG samples calculated were both close to 1 (Fig. S5), indicating a high RNA-seq data reliability.

We deposited the raw sequence reads into the National Center for Biotechnology Information (NCBI) Short Read Archive (SRA) repository with the accession numbers SRP065638 and SRA308886.

### Seed germination assays

Seed germination assays under salt stress conditions and ABA treatment were performed as described previously [15]. The numbers of germinated seeds and green seedlings were counted on the fourth day. Each assay was repeated three times.

### Leaf electrolyte leakage, chlorophyll, relative water contents and photosynthesis parameters

Measurements of Leaf electrolyte leakage (EL), chlorophyll and relative water content (RWC) were conducted as described previously [133, 139]. Each measurement was conducted in three replicates.

### Statistical analysis

The data were analyzed by Microsoft excel 2010 (Microsoft, USA) and significance of differences between data sets was evaluated by SAS software 9.2 (SAS institute Inc. USA)

## Supplementary information

**Additional file 1: Table S1.** List of assembled transcripts up-regulated or down-regulated (log2FC > 1 or < − 1, FDR < 0.01) in AsHSP26.8a transgenic (TG) *Arabidopsis*, relative to wild type (WT) plants.

**Additional file 2: Table S2.** Primers used in this study.

**Additional file 3: Figure S1.** AsHSP26.8a nucleic acid sequence (**A**) and amino acid sequence (**B**) alignment with AsHSP26.8. **Figure S2.** Amino acid sequence alignment of AsHSP26.8a and other plant chloroplast localized (CP) sHSPs. Accession umbers of sHSPs are: *Agrostis stolonifera* HSP26.8a (KU353578); *Aegilops kotschyi* HSP26.8 (CAI96511); *Triticum dicoccoides* HSP26.4 (CAI96512); *Hordeum vulgare* HSP26 (AAB28590); *Brachypodium distachyon* sHSP (XP_003558381.1); *Spartina alterniflora* HSP27.1 (AFP96756.1); *Zea mays* HSP18 (CAM12752.1); *Oryza sativa* HSP26 (BAA78385.1). **Figure S3.** The phylogenetic relationship of the AsHSP26.8a with different classes of sHSPs from other plant species. Accession numbers of the sHSPs are: *Arabidopsis thaliana* HSP17.4 (X17293); *Arabidopsis thaliana* HSP17.6 (X16076); *Oryza sativa* HSP17.4 (D12635); *Daucus carota* HSP18.0 (X53852); *Helianthus annuus* HSP17.6 (X59701); *Agrostis stolonifera* HSP17 (KT272405); *Triticum aestivum* HSP16.9A (X13431); *Oryza sativa* HSP16.9A (X60820); *Zea mays* HSP16.9 (X65725); *Solanum lycopersicum* HSP17.8 (X56138); *Glycine max* HSP17.5 (M11318); *Glycine max* HSP17.6 (MI1317); Medicago sativa HSP18.1 (X58710); *Pisum sativum* HSP18.1 (M33899); *Arabidopsis thaliana* HSP22 (U11501); *Glycine max* HSP22 (X63198); *Pisum sativum* HSP22 (M33898); *Arabidopsis thaliana* HSP18.5 (816448); *Oryza sativa* HSP17.6 (113595340); *Arabidopsis thaliana* HSP15.7 (833746); *Arabidopsis thaliana* HSP26.5 (841687); *Oxybasis rubra* HSP23 (X15333); *Arabidopsis thaliana* HSP23.6 (828623); *Oryza sativa* HSP22 (4330786); *Agrostis stolonifera* HSP26.8a (KU353578); *Triticum aestivum* HSP26.6 (X58280); *Zea mays* HSP26 (L28712); Petunia x hybrida HSP21 (X54103); *Arabidopsis thaliana* HSP21 (X54102); *Glycine max* HSP22 (X07188); *Pisum sativium* HSP21 (X07187); *Arabidopsis thaliana* HSP15.4 (828276); *Oryza sativa* HSP18.8 (4343386); *Arabidopsis thaliana* HSP17.4 (841843); *Oryza sativa* HSP17.6B (4330933); *Lilium longiflorum* HSP17.6 (D21816); *Zea mays* HSP17.5 (X54076); *Zea mays* HSP17.8 (X54075); *Triticum aestivum* HSP17.3 (X58279); *Arabidopsis thaliana* HSP17.6 (X63443); *Ipomoea nil* HSP18.8 (M99430); *Ipomoea nil* HSP17.2 (M99429); *Glycine max* HSP17.9 (X07159); *Pisum sativum* HSP17.7 (M33901); *Arabidopsis thaliana* HSP21.7 (835555); *Oryza sativa* HSP22.2 (4339231). **Figure S4.** Gene ontology (GO) classification of the annotated unigenes or differentially expressed genes (DEGs). Unigenes with best BLAST hits were aligned to GO database. A total of 26,326 unigenes (red) and 261 DEGs (blue) were assigned to at least one GO term and grouped into three main GO categories (cellular component, molecular function and biological process) and 53 GO terms. Left Y-axis represents the percentages of unigenes or DEGs in each main category. Right Y-axis indicates the numbers of unigenes or DEGs in each GO term. **Figure S5.** Correlation assessment of a pair of biological replicates in wild type (WT) and AsHSP26.8a transgenic (TG) plants, respectively, used for RNA-seq. **A,** Pearson’s correlation coefficient between the two biological replicates of the wild type (E01 and E02) or AsHSP26.8a transgenic plants (E05 and E06). **B** and **C**, Correlation plots of a pair of biological replicates from wild type (E01 and E02), (**B**) and AsHSP26.8a transgenic line TG3 (E05 and E06) (**C**), respectively. Each dot in the plot represents a gene, denoting the log10 of the gene expression (FPKM) in the two replicate samples (x-axis and y-axis). The closer is the dot to the diagonal, the smaller is the difference in expression for the corresponding gene in the two replicates.

## Data Availability

All relevant data are included in this article and its supplementary information files.

## Abbreviations

CaM: Calmodulins
CaMV35S: Cauliflower mosaic virus 35S;
CBLs: Calcineurin B-like proteins (CBLs)
CDPKs: Calcium dependent protein kinases
CMLs: Calmodulin-like proteins
DEGs: Differentially expressed genes
EL: Electrolyte leakage
FDR: False discovery rate
GFP: Green florescent protein
GO: Gene Ontology
HSFs: Heat shock transcription factors
HSPs: Heat shock proteins
LEA: Late embryogenesis abundant
MW: Molecular weight
RNA-seq: RNA sequencing
RWC: Relative water content
TF: Transcription factor
