# Supplementary material for: AsHSP26.8a, a creeping bentgrass small heat shock protein integrates different signaling pathways to modulate plant abiotic stress response
Source: BMC Plant Biol. 2020 Apr 28;20:184. doi: 10.1186/s12870-020-02369-5 (PMC7189581; doi:10.1186/s12870-020-02369-5)
Supplement: Supplementary file 1 — Additional file 1: Table S1. List of assembled transcripts up-regulated or down-regulated (log2FC > 1 or < − 1, FDR < 0.01) in AsHSP26.8a transgenic (TG) Arabidopsis, relative to wild type (WT) plants. [file 12870_2020_2369_MOESM1_ESM.pptx]

## Slide 1
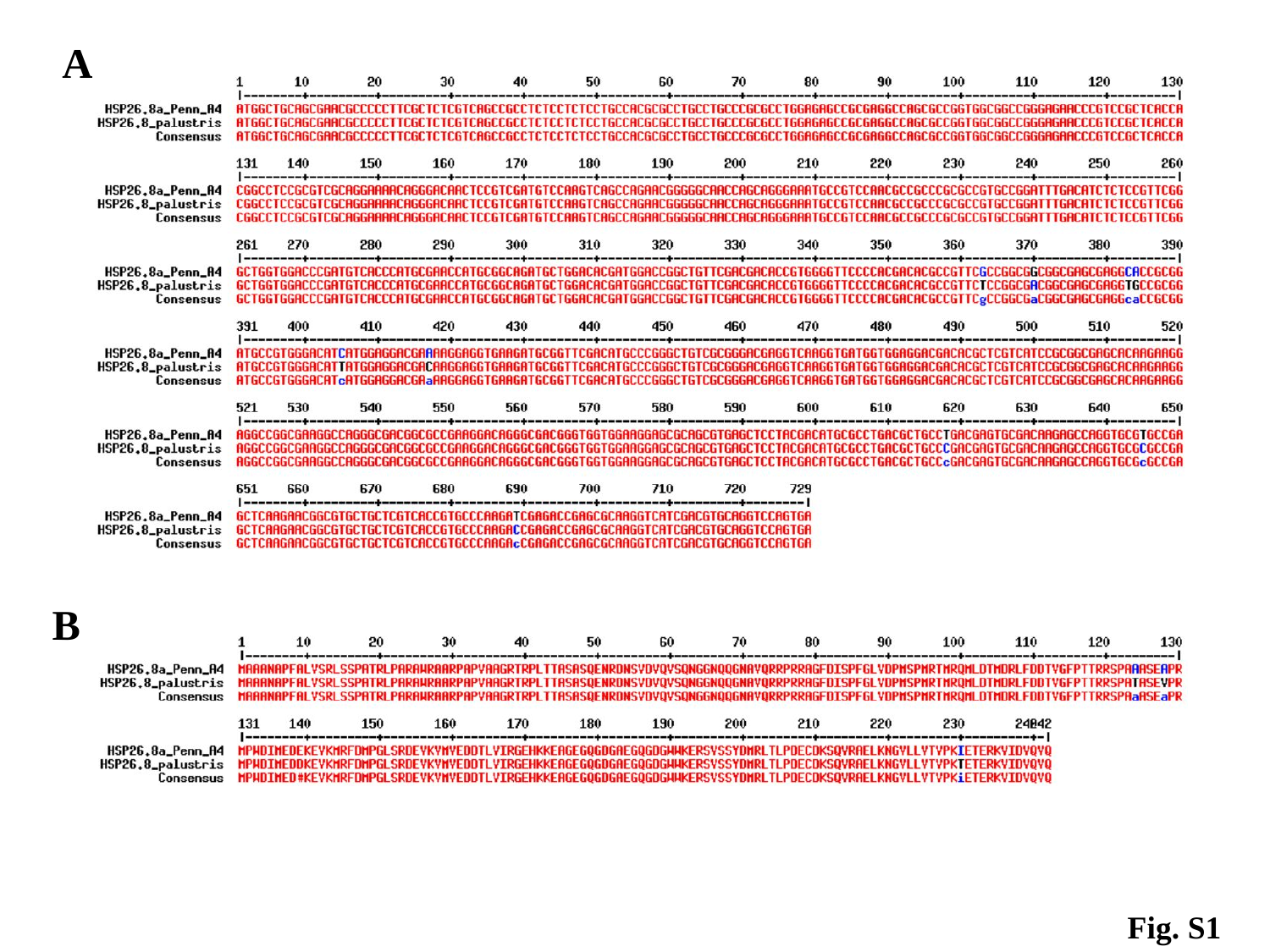

A
B
Fig. S1

## Slide 2
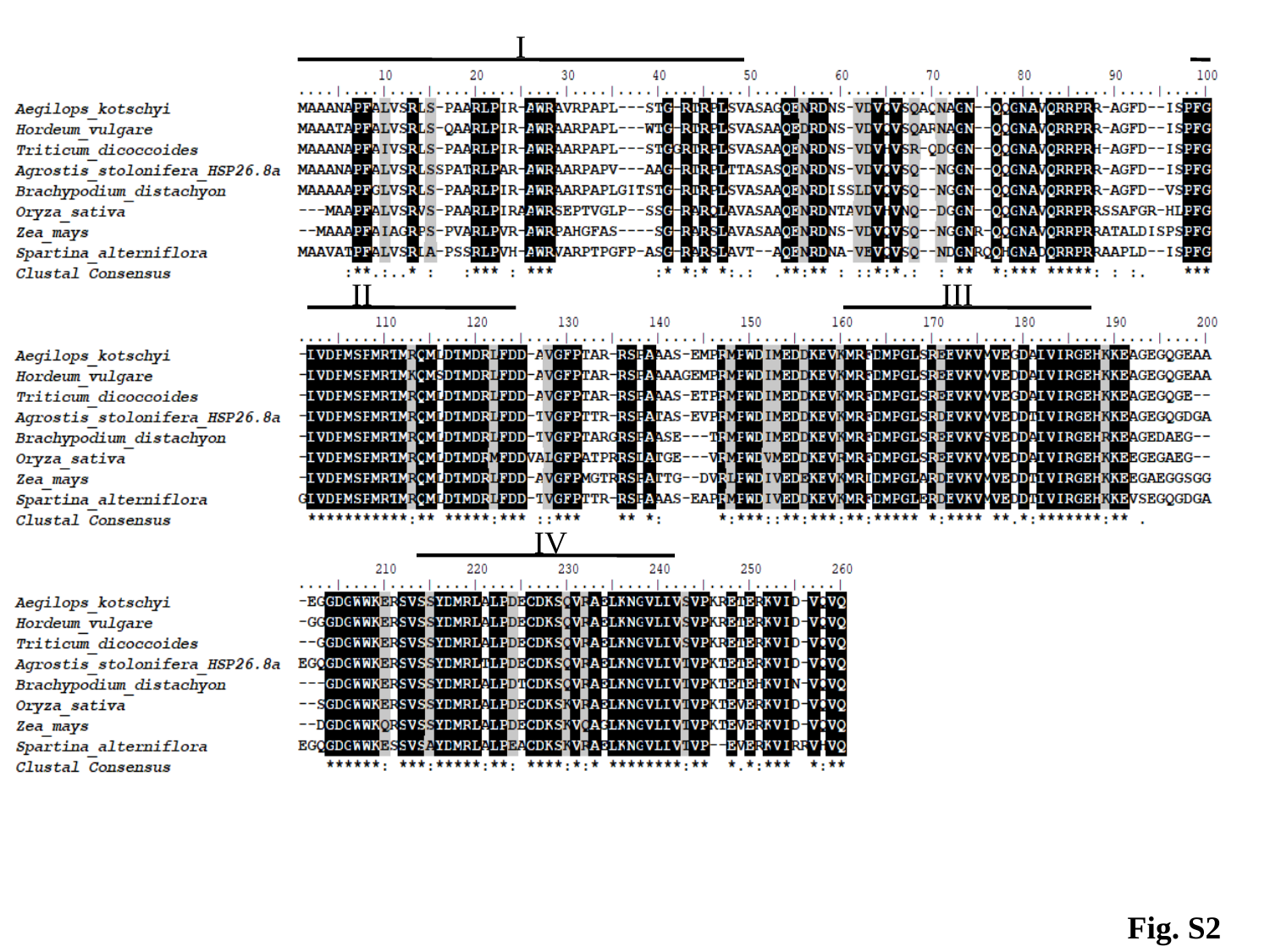

I
II
III
IV
Fig. S2

## Slide 3
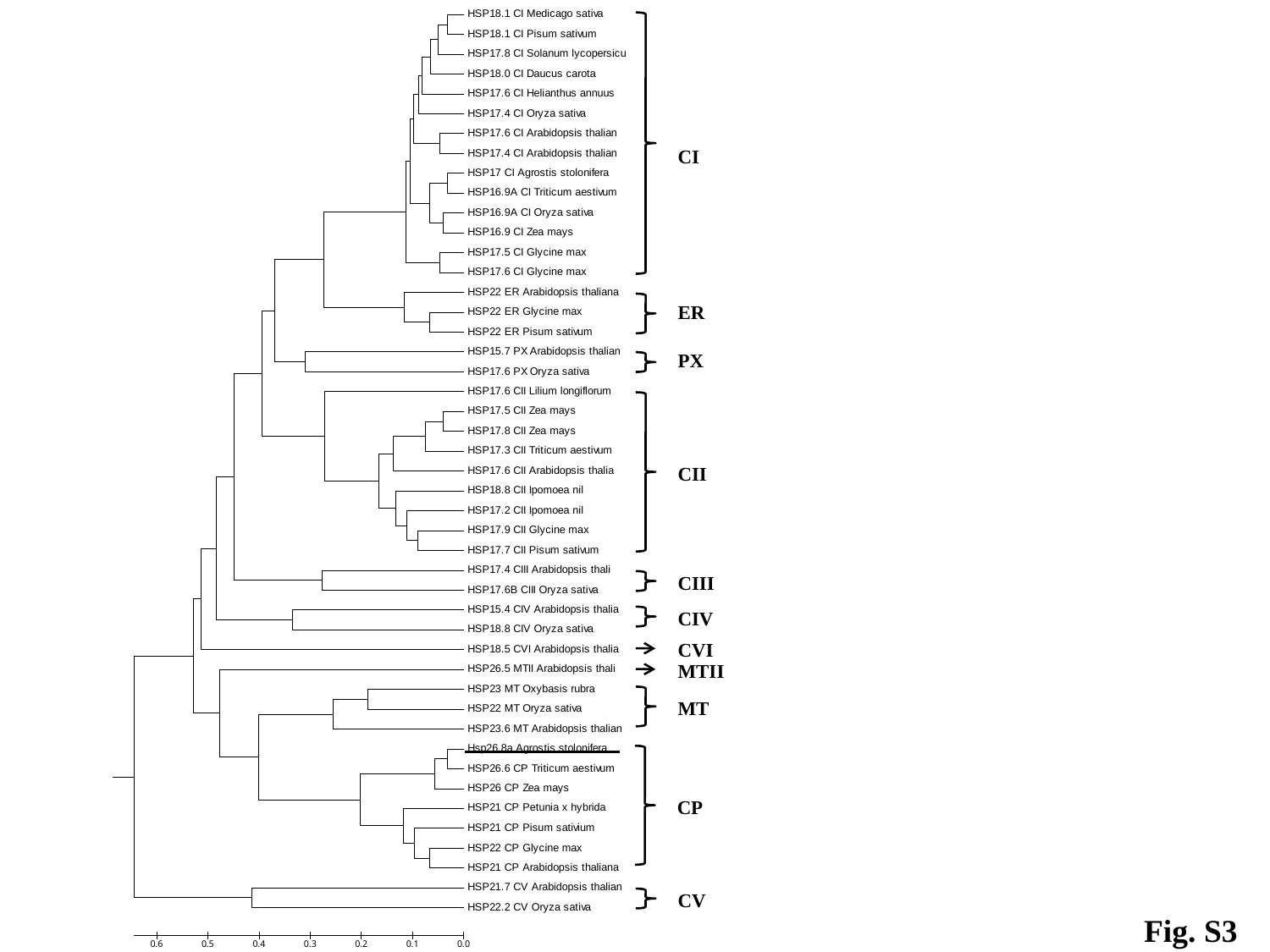

CI
ER
PX
CII
CIII
CIV
CVI
MTII
MT
CP
CV
Fig. S3

## Slide 4
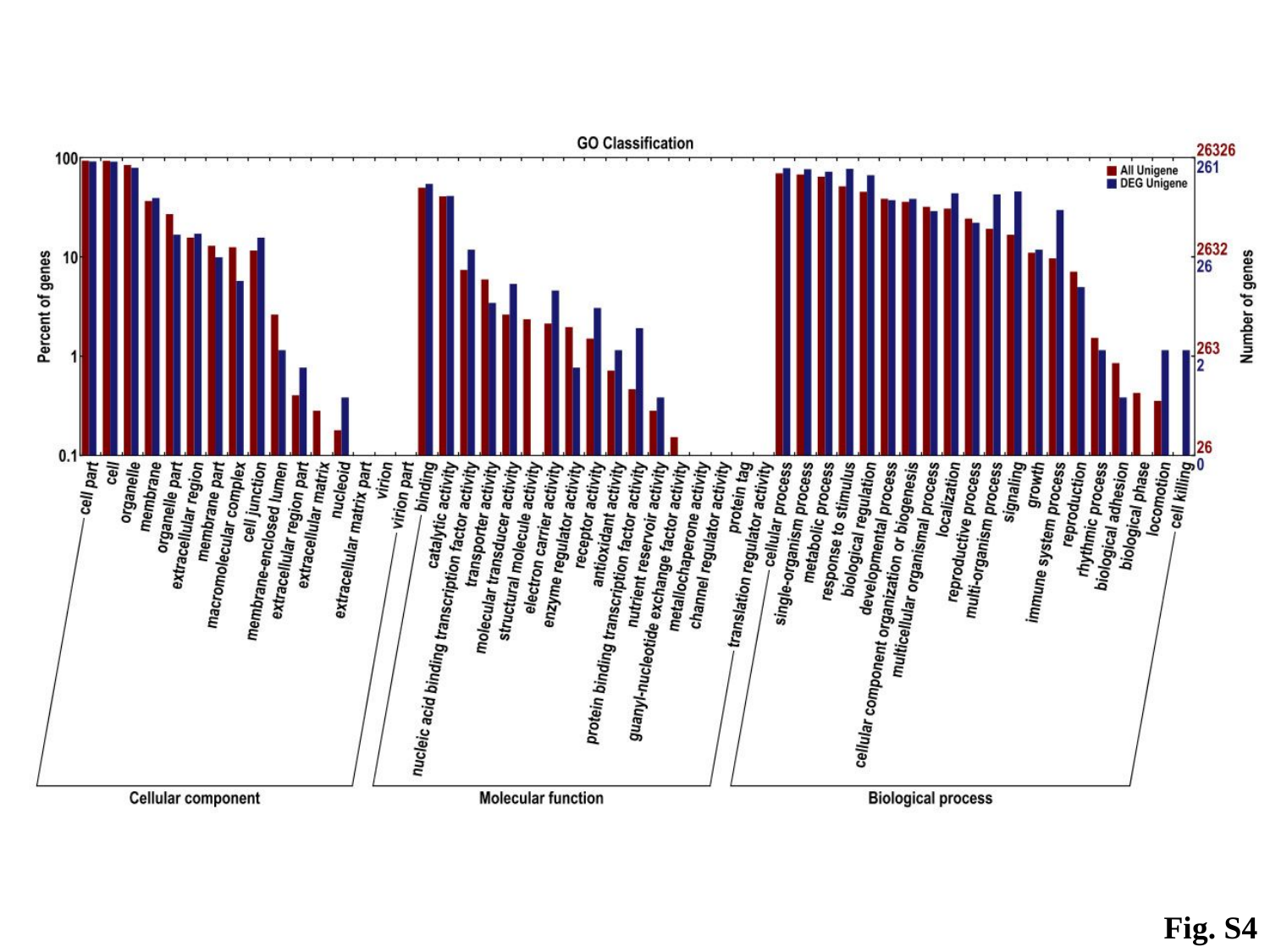

Fig. S4

## Slide 5
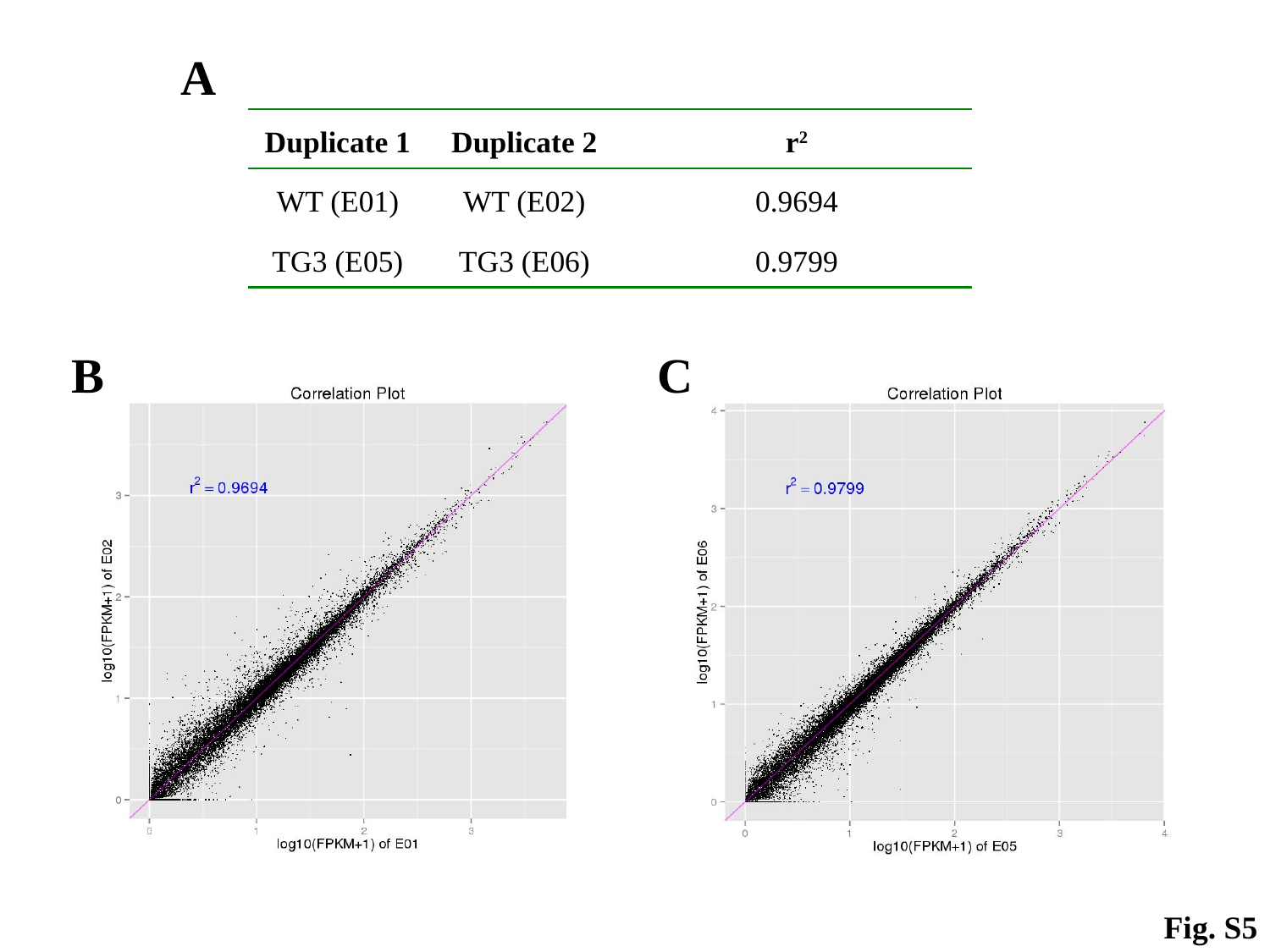

A
| Duplicate 1 | Duplicate 2 | r2 |
| --- | --- | --- |
| WT (E01) | WT (E02) | 0.9694 |
| TG3 (E05) | TG3 (E06) | 0.9799 |
B
C
Fig. S5
