# Supplementary material for: AsHSP26.8a, a creeping bentgrass small heat shock protein integrates different signaling pathways to modulate plant abiotic stress response
Source: BMC Plant Biol. 2020 Apr 28;20:184. doi: 10.1186/s12870-020-02369-5 (PMC7189581; doi:10.1186/s12870-020-02369-5)
Supplement: Supplementary file 3 — Additional file 3: Figure S1. AsHSP26.8a nucleic acid sequence (A) and amino acid sequence (B) alignment with AsHSP26.8. Figure S2. Amino acid sequence alignment of AsHSP26.8a and other plant chloroplast localized (CP) sHSPs. Accession umbers of sHSPs are: Agrostis stolonifera HSP26.8a (KU353578); Aegilops kotschyi HSP26.8 (CAI96511); Triticum dicoccoides HSP26.4 (CAI96512); Hordeum vulgare HSP26 (AAB28590); Brachypodium distachyon sHSP (XP_003558381.1); Spartina alterniflora HSP27.1 (AFP96756.1); Zea mays HSP18 (CAM12752.1); Oryza sativa HSP26 (BAA78385.1). Figure S3. The phylogenetic relationship of the AsHSP26.8a with different classes of sHSPs from other plant species. Accession numbers of the sHSPs are: Arabidopsis thaliana HSP17.4 (X17293); Arabidopsis thaliana HSP17.6 (X16076); Oryza sativa HSP17.4 (D12635); Daucus carota HSP18.0 (X53852); Helianthus annuus HSP17.6 (X59701); Agrostis stolonifera HSP17 (KT272405); Triticum aestivum HSP16.9A (X13431); Oryza sativa HSP16.9A (X60820); Zea mays HSP16.9 (X65725); Solanum lycopersicum HSP17.8 (X56138); Glycine max HSP17.5 (M11318); Glycine max HSP17.6 (MI1317); Medicago sativa HSP18.1 (X58710); Pisum sativum HSP18.1 (M33899); Arabidopsis thaliana HSP22 (U11501); Glycine max HSP22 (X63198); Pisum sativum HSP22 (M33898); Arabidopsis thaliana HSP18.5 (816448); Oryza sativa HSP17.6 (113595340); Arabidopsis thaliana HSP15.7 (833746); Arabidopsis thaliana HSP26.5 (841687); Oxybasis rubra HSP23 (X15333); Arabidopsis thaliana HSP23.6 (828623); Oryza sativa HSP22 (4330786); Agrostis stolonifera HSP26.8a (KU353578); Triticum aestivum HSP26.6 (X58280); Zea mays HSP26 (L28712); Petunia x hybrida HSP21 (X54103); Arabidopsis thaliana HSP21 (X54102); Glycine max HSP22 (X07188); Pisum sativium HSP21 (X07187); Arabidopsis thaliana HSP15.4 (828276); Oryza sativa HSP18.8 (4343386); Arabidopsis thaliana HSP17.4 (841843); Oryza sativa HSP17.6B (4330933); Lilium longiflorum HSP17.6 (D21816); Zea mays HSP17.5 (X54076); Zea mays HSP17.8 (X [file 12870_2020_2369_MOESM3_ESM.doc]

**Supporting information**

**Supplemental Table S1.** List of assembled transcripts up-regulated or down-regulated (log2FC>1 or <-1, FDR<0.01) in AsHSP26.8a transgenic (TG) *Arabidopsis*, relative to wild type (WT) plants.

**Supplemental Table S2.** Primers used in this study.

AsHSP26.8a cloning and RT-PCR for gene expression

F: 5'- AGG ATC CAT GGC TGC AGC GAA CGC CCC CTT C-3'

R: 5'- GGG ATC CTC ACT GGA CCT GCA CGT CGA TGA CC-3'

bar RT-PCR

F: 5'-GTC TGC ACC ATC GTC AAC CAC TAC-3'

R: 5'- GTC CAG CTG CCA GAA ACC CAC-3'

qRT-PCR for genes in *Arabidopsis*

| AtActin1 | Forward | 5'-CCG ACA GAA TGA GCA AAG AGA-3' |
| --- | --- | --- |
| AtActin1 | Reverse | 5'-CCT CCA ATC CAG ACA GAG TAT TT-3' |
| AtTUB6 | Forward | 5'-TGGTACACAGGTGAAGGAATG-3' |
| AtTUB6 | Reverse | 5'-GCAGTTGCGTCTTGGTATTG-3' |
| AtDREB1B | Forward | 5'-CTG AAA TGT TTG GCT CCG ATT AC-3 |
| AtDREB1B | Reverse | 5'-ACG AGT CTC ACG AAA CTT CTT AC-3 |
| AtERF105 | Forward | 5'- CAACTCTAAGCCAACGCAAAC -3' |
| AtERF105 | Reverse | 5'-TCTCCTGACGCCTCTGTAAT-3' |
| AtHSFB2a | Forward | 5'-GCCCACTGATCGGAGTTATT -3' |
| AtHSFB2a | Reverse | 5'-CGGACTCGCTTCTTCTTCTT-3' |
| AtHSFC1 | Forward | 5'-GCAGATGATGGCGTTTCTTTAC-3' |
| AtHSFC1 | Reverse | 5'- GTTGCTTTGTCCGTTCTTTCTC -3' |
